# Applying low coverage whole genome sequencing to detect malignant ovarian mass

**DOI:** 10.1186/s12967-021-03046-3

**Published:** 2021-08-26

**Authors:** Ming Chen, Pengqiang Zhong, Mengzhi Hong, Jinfeng Tan, Xuegao Yu, Hao Huang, Juan Ouyang, Xiaoping Lin, Peisong Chen

**Affiliations:** 1 Department of Gynecology, The First Affiliated Hospital, Sun Yat-Sen University, Guangzhou, China; 2 Department of Clinical Laboratory, Department of Laboratory Medicine, The First Affiliated Hospital of Sun Yat-Sen University, 58 Zhongshan road II, Guangzhou, Guangdong People’s Republic of China; 3 Department of Nuclear Medicine, Sun Yat-Sen University Cancer Center, 651 Dongfengdong Road, Guangzhou, Guangdong People’s Republic of China; 4 State Key Laboratory of Oncology in South China, Collaborative Innovation Center for Cancer Medicine, Sun Yat-Sen University Cancer Center, 651 Dongfengdong Road, Guangzhou, Guangdong People’s Republic of China

**Keywords:** Ovarian cancers, Whole genome sequencing, ROMA, RM, ROC

## Abstract

To evaluate whether low coverage whole genome sequencing is suitable for the detection of malignant pelvic mass and compare its diagnostic value with traditional tumor markers. We enrolled 63 patients with a pelvic mass suspicious for ovarian malignancy. Each patient underwent low coverage whole genome sequencing (LCWGS) and traditional tumor markers test. The pelvic masses were finally confirmed via pathological examination. The copy number variants (CNVs) of whole genome were detected and the Stouffers Z-scores for each CNV was extracted. The risk of malignancy (RM) of each suspicious sample was calculated based on the CNV counts and Z-scores, which was subsequently compared with ovarian cancer markers CA125 and HE4, and the risk of ovarian malignancy algorithm (ROMA). Receiver Operating Characteristic Curve (ROC) were used to access the diagnostic value of variables. As confirmed by pathological diagnosis, 44 (70%) patients with malignancy and 19 patients with benign mass were identified. Our results showed that CA125 and HE4, the CNV, the mean of Z-scores (Zmean), the max of Z-scores (Zmax), the RM and the ROMA were significantly different between patients with malignant and benign masses. The area under curve (AUC) of CA125, HE4, CNV, Zmax, and Zmean was 0.775, 0.866, 0.786, 0.685 and 0.725 respectively. ROMA and RM showed similar AUC (0.876 and 0.837), but differed in sensitivity and specificity. In the validation cohort, the AUC of RM was higher than traditional serum markers. In conclusion, we develop a LCWGS based method for the identification of pelvic mass of suspicious ovarian cancer. LCWGS shows accurate result and could be complementary with the existing diagnostic methods.

**Supplementary Information:**

The online version contains supplementary material available at 10.1186/s12967-021-03046-3.

## Introduction

According to the latest 2018 global cancer data report, the incidence of ovarian tumors in female reproductive system accounted for 3.4% of all female tumors in China, and the number of women who died of malignant ovarian tumors accounted for 4.4% of all female patients who died of tumors [1]. Ovarian cancer has become the second highest incidence and mortality of female reproductive system tumor following cervical cancer [1, 2]. Because of the small size of the ovary and its position in the pelvic cavity, ovarian tumor itself lacks typical symptoms in early stage [3]. Patients often find that they have ovarian tumor after the pelvic cavity has a huge mass or bleeding in the vagina [4, 5]. At this time, the tumor has developed to the late stage and most of them spread to other pelvic organs, and has missed the best time for treatment [6]. Therefore, the early detection of ovarian tumors is critical for clinical management and prognosis of patients. Multiple efforts have been made to evaluate traditional markers including serum concentration of CA125 and HE4 in the screening of ovarian cancers [7]. However, these markers did not meet the standards required to advocate population-based screening regarding with the diagnostic sensitivity and or specificity [8, 9]. In order to improve the accuracy of diagnosis for ovarian cancer, additional cancer-specific diagnostic methods may be required.

In recent years, the rapid development in the field of next generation sequencing (NGS) and its application in low coverage whole genome sequencing (LCWGS) makes the detection of tumor-specific copy number alterations (CNA) in cell-free DNA feasible [10, 11]. Evidence has showed that tumor-derived chromosome abnormalities would be detectable in the plasma of patients prior to surgery [10, 12].

Previous studies have reported that occult pelvic cancers can be detected by LCWGS testing but it might cause false positive results [13]. However, the diagnostic accuracy of LCWGS platform and analytic pipeline for ovarian cancer remains unknown. The aim of this study is to investigate whether a clinical LCWGS platform could detect ovarian cancers in patients with pelvic masses based on the abnormal plasma DNA copy number variants (CNVs), and to compare the diagnostic accuracy with traditional screening markers including CA125 and HE4, and the score of risk of ovarian malignancy algorithm (ROMA) [14].

## Methods

### Subjects and samples

Sixty-three patients with a pelvic mass suspicious for ovarian malignancy, who were referred to the gynecology department of the First Affiliated Hospital of Sun Yat-sen university from January 2018 to July 2019 were recruited in this study. In addition, a cohort of 39 healthy female individuals were also recruited. Blood samples were collected using EDTA anticoagulated tube and sent for laboratory within 2 h. Another 24 cases from Sun Yat-Sen University Cancer Center from June 2021 to July 2021 were enrolled into the validation cohorts and used to validate our results. The study approval was obtained from the ethical committee of the First Affiliated Hospital of Sun Yat-sen university (S/55904). All participants submitted their written informed consents.

### Sample processing and LCWGS

The blood samples were firstly centrifuged at 1600 g for ten minutes at 4 ℃, and then the supernatant was centrifuged at 16,000 g again for ten minutes at 4 ℃. The plasma was stored − 80 °C until analysis. The isolation, purification, library construction and sequencing of cell free DNA from the blood were performed by using a Fetal Aneuploidies Trisomy Detection Kit (Daan Gene Corp, China) on Ion Proton next-generation sequencer (Life Technologies) which was certified by the China Food and Drug Administration. All procedures were performed according to the manufacture’s protocol.

### Bio-informatics analysis

Raw sequencing reads were mapped to the human reference genome Hg19 using BWA (v0.7.1). Duplicate and low-quality reads were removed by Picard Tools (v1.11) and Samtools (v0.1.18) respectively. TorrentSuit software (v3.6) and a NIPT-plus plugin (provided by the Daan Gene Corp) was used to calculate the Stouffers Z-scores for whole chromosomes and CNV ≥ 5.0 MB. |Z-scores|> = 3 were marked as high risk. Both CNV counts and |Z-scores| _(>=3)_ were extracted from each sample for further analysis.

### Analysis of malignant risk

For further analysis of the risk of malignancy, data from 39 healthy females was used to form a baseline. Firstly, we calculated the mean of CNV counts and |Z-scores| (≥3), then the risk of malignancy(RM) of each suspicious sample was calculated as (CNV counts _suspiciou**s**_- CNV counts _mean of healthy_) X (|Z-scores| _suspiciou**s**_- |Z-scores| _mean of healthy_).

### Tumor marker detection and ROMA scores

HE4 and CA125 were tested in stored plasma using the ARCHITECT HE4 and CA125 assays (Abbott Diagnostics, Abbott Park, IL, USA) according to the manufacturer’s instructions.

### Pathology diagnosis of pelvic mass

All diagnoses of patients were confirmed via pathological examination by pathologists who were blind to the results of clinical laboratory testing. Tumor staging was performed according to the International Federation of Gynecology and Obstetrics (FIGO) criteria (2010).

### Statistical analysis

Statistical analysis was carried out by an online statistics tool (http://dxonline.deepwise.com/) and R software (Version 4.0.1) with pROC and Rattle package (5–7). Receiver operating characteristics (ROC) curve was used to evaluate the diagnostic value. A two-tailed *P* value of less than 0.05 was considered statistically significant.

## Results

### Clinical and pathology data of subjects

This study included 63 patients with a pelvic mass suspicious of ovarian malignancy, who were finally identified as 34 (54%) high grade malignancy, 10 (16%) low grade malignancy and 19 (30%) benign mass by pathological diagnosis. The median age of premenopausal patients were 35 years (range, 16–53 years), and the median age of postmenopausal patients were 62 years (range, 46–83 years). The median age of patients with malignancies was 51 years (range: 21–70) and that of benign diseases was 30 years (range: 18–52). There was a significant difference in age distribution between these 2 groups of patients (*P* < 0.01). The FIGO stage of ovarian cancers patients included 13 (30%) I stage, 6 (14%) II stage, 18 (41%) III stage and 7 (16%) IV stage. The clinical and pathological data of subjects were listed in Table 1.

**Table 1:** Clinical and pathology data of subjects

Group	Pathology	Number	Ratio
High grade (34)	Serous cystadenocarcinoma	18	53%
	Mucinous cystadenocarcinoma	8	24%
	Clear cell carcinoma	3	9%
	Yolk sac tumor	1	3%
	Adenoid carcinoma of endometrium	2	6%
	Granular cell carcinoma	1	3%
Low grade (10)	Borderline serous cystadenoma	4	40%
	Borderline mucinous cystadenoma	3	30%
	Borderline endometrial carcinoma	2	20%
Benign (19)	Chocolate cyst	9	47%
	Teratoma	5	26%
	Mucinous cystadenoma	3	16%
	Spindle cell tumor	1	5%
	Tuberculous granuloma	1	5%
Age (median, range)			
35 (16-53)	Premenopausal	23	35%
62 (46-83)	Postmenopausal	40	63%
**FIGO stage**			
Ovarian cancers	I	13	30%
	II	6	14%
	III	18	41%
	IV	7	16%

### LCWGS on CNVs

**Fig. 1:**
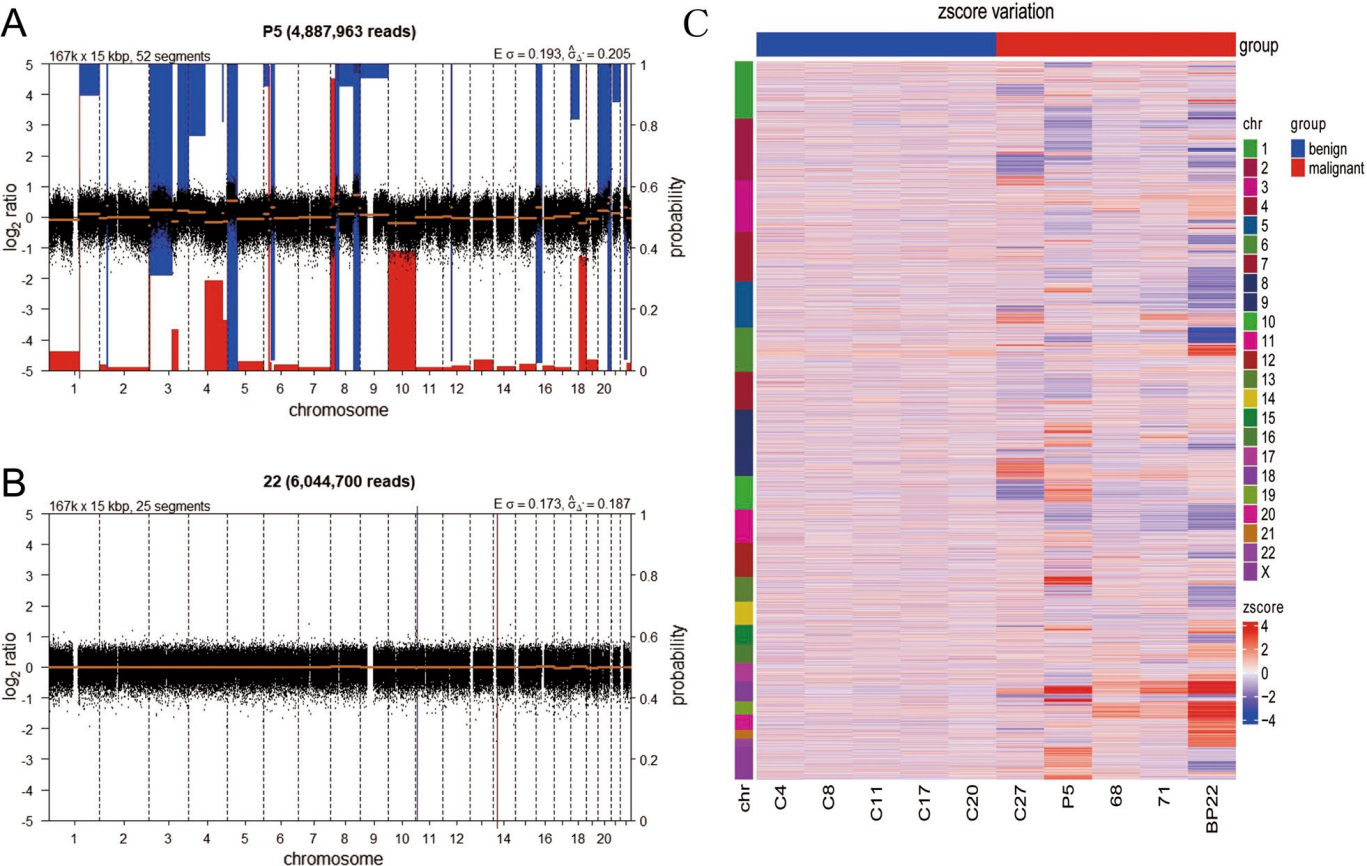
A representative figure for ovarian cancer and benign disease.** A** NIPT results from a patient with FIGO Stage III Serous cystadenocarcinoma which showed multiple regions of copy number variants. The blue regions indicate duplications while the red regions indicate deletions. **B** NIPT results from a patient with Teratoma. No copy number variants was found in this patient. **C** A heat map to show the difference of CNVs in each chromosome between benign and malignant patients

**Fig. 2:**
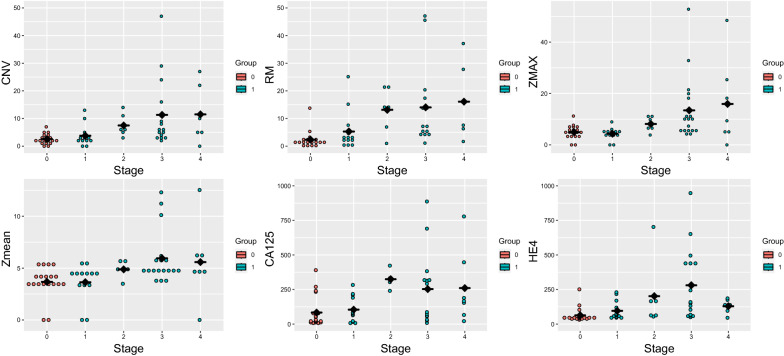
CNV, RM, ZMAX, Zmean, CA125 and HE4 in different FIGO stage

LCWGS used a whole genome low coverage strategy to analyze the CNVs. For each sample, more than 5 M (5.9 ± 0.68 for all samples) reads was obtained. The coverage of each sample is about 0.35 × . A representative LCWGS figure for ovarian cancer and benign disease was shown in Fig. 1. The results from a patient with FIGO Stage III serous cystadenocarcinoma showed multiple regions of CNV (Fig. 1A). And the results from a patient with teratoma showed that no CNV (Fig. 1B). In this study, only 7 patients with malignancy showed trisomy or monosomy as indicated by LCWGS. To further investigate the diagnostic performance of LCWGS, CNV counts, max of Z scores (Zmax) of all CNVs, mean of Z scores (Zmean) and RM was calculated from each sample. Significant difference of LCWGS based index was found between patients with malignant and benign tumors. We have provided all the CNVs in supplement data (Additional file 1: Supplement Table 1 and Additional file 2: Supplement Table 2). However, it is difficult to identify the specific CNVs at the resolution of 5 MB or display all the results in one figure. So we selected 10 samples to generate a heat map to show the difference of CNVs in each chromosome between benign and malignant patients (Fig. 1C). Patients with malignancy showed higher level in LCWGS based index than patients with benign disease. In addition, these indexes were closely related to different FIGO stage (Fig. 2). The positive rates of RM in Stage I, Stage II, Stage III and Stage IV was 76%, 83%, 94% and 100% respectively.

### Traditional tumor markers

The serum concentration of CA125 was 416.457 ± 747.887 U/ml (Mean ± SD), HE4 was 219.192 ± 457.614 U/ml and ROMA was 0.534 ± 0.422 in all subjects. There were significant differences between the concentration of CA125(560.282 ± 854.994 VS 83.387 ± 112.353, U/ml) and HE4(286.382 ± 534.32 VS 63.595 ± 51.849, U/ml) in patients with malignant and benign diseases. Besides, menopausal status was significant correlation with malignant and benign diseases (Table 2).

**Table 2:** Comparison of laboratory index

Group	Counts	Mean±SD	Median (0.25-0.75)	Median (0.025–0.975)	Min	Max	CV	*P*
AGE (years)								
Benign mass	19	34.474±10.808	30 (27–41.5)	30 (18.05–52.4)	14	56	31.35%	0.001**
Malignancy mass	44	48.25±13.404	51.5 (39–56.25)	51.5 (21.075–70.85)	18	73	27.78%	
Total	63	44.095±14.112	46 (35–55)	46 (19.65–69.9)	14	73	32.00%	
CA125 (U/ml)								
Benign mass	19	83.387±112.353	24.89 (13.765–78.24)	24.89 (7.425–336.055)	6.98	390.1	134.74%	0.001**
Malignancy mass	44	560.282±854.994	214.7 (65.523–428.975)	214.7 (8.025–2986.7)	6.98	3190.6	152.60%	
Total	63	416.457±747.887	86.62 (25.445–318.2)	86.62 (7.524–2756.8)	6.98	3190.6	179.58%	
HE4 (U/ml)								
Benign mass	19	63.595±51.849	45.49 (40.175–53.49)	45.49 (34.13–199.065)	31.7	251.4	81.53%	0.000**
Malignancy mass	44	286.382±534.32	128.55 (60.925–220.55)	128.55 (45.6–1458.577)	45.49	3235.9	186.58%	
Total	63	219.192±457.614	67.8 (46.71–167.65)	67.8 (38.206–1196.235)	31.7	3235.9	208.77%	
ROMA								
Benign mass	19	0.189±0.278	0.06 (0.045–0.13)	0.06 (0.034–0.905)	0.03	0.99	147.09%	0.000**
Malignancy mass	44	0.683±0.387	0.96 (0.277–1)	0.96 (0.06–1)	0.06	1	56.66%	
Total	63	0.534±0.422	0.52 (0.075–1)	0.52 (0.04–1)	0.03	1	79.03%	
CNV								
Benign mass	19	2.579±1.805	2 (1.5–3.5)	2 (0–6.1)	0	7	69.99%	0.000**
Malignancy mass	44	14.432±27.812	5 (3–11.5)	5 (0–69.575)	0	168	192.71%	
Total	63	10.857±23.822	4 (2–8.5)	4 (0–60.55)	0	168	219.42%	
Zmax								
Benign mass	19	4.937±2.6	5.06 (3.445–6.36)	5.06 (0–9.623)	0	11.22	52.66%	0.021*
Malignancy mass	44	10.421±11.091	6.05 (4.672–10.973)	6.05 (0–47.258)	0	52.77	106.43%	
Total	63	8.767±9.681	5.66 (4.455–9.745)	5.66 (0–39.833)	0	52.77	110.43%	
Zmean								
Benign mass	19	3.676±1.499	3.83 (3.305–4.305)	3.83 (0–5.429)	0	5.51	40.78%	0.005**
Malignancy mass	44	5.059±2.536	4.77 (4.245–5.66)	4.77 (0–12.228)	0	12.53	50.13%	
Total	63	4.642±2.35	4.46 (3.69–5.265)	4.46 (0–11.71)	0	12.53	50.62%	
RM								
Benign mass	19	0.879±2.304	0.32 (− 0.04-0.84)	0.32 (− 0.683–6.699)	− 0.71	9.79	262.12%	0.000**
Malignancy mass	44	63.872±216.976	4.73 (1.532–16.795)	4.73 (− 0.139–608.251)	− 0.15	1289.65	339.70%	
Total	63	44.874±183.035	2.39 (0.185–10.185)	2.39 (− 0.502–468.421)	− 0.71	1289.65	407.89%	

	Benign mass (N = 19)	Malignancy mass (N = 44)	Total (N = 63)	X²	*P*
Marriage					
Yes	16 (84.2%)	40 (90.9%)	56 (88.9%)	0.115	0.734
No	3 (15.8%)	4 (9.1%)	7 (11.1%)		
Childbirth					
Yes	14 (73.7%)	40 (90.9%)	54 (85.7%)	1.962	0.161
No	5 (26.3%)	4 (9.1%)	9 (14.3%)		
Menopausal status					
PreMenopausal	18 (94.7%)	22 (50.0%)	40 (63.5%)	11.457	0.001**
PostMenopausal	1 (5.3%)	22 (50.0%)	23 (36.5%)		

*p<0.05, **p<0.01

### Correlation between traditional tumor markers and LCWGS index

**Fig. 3:**
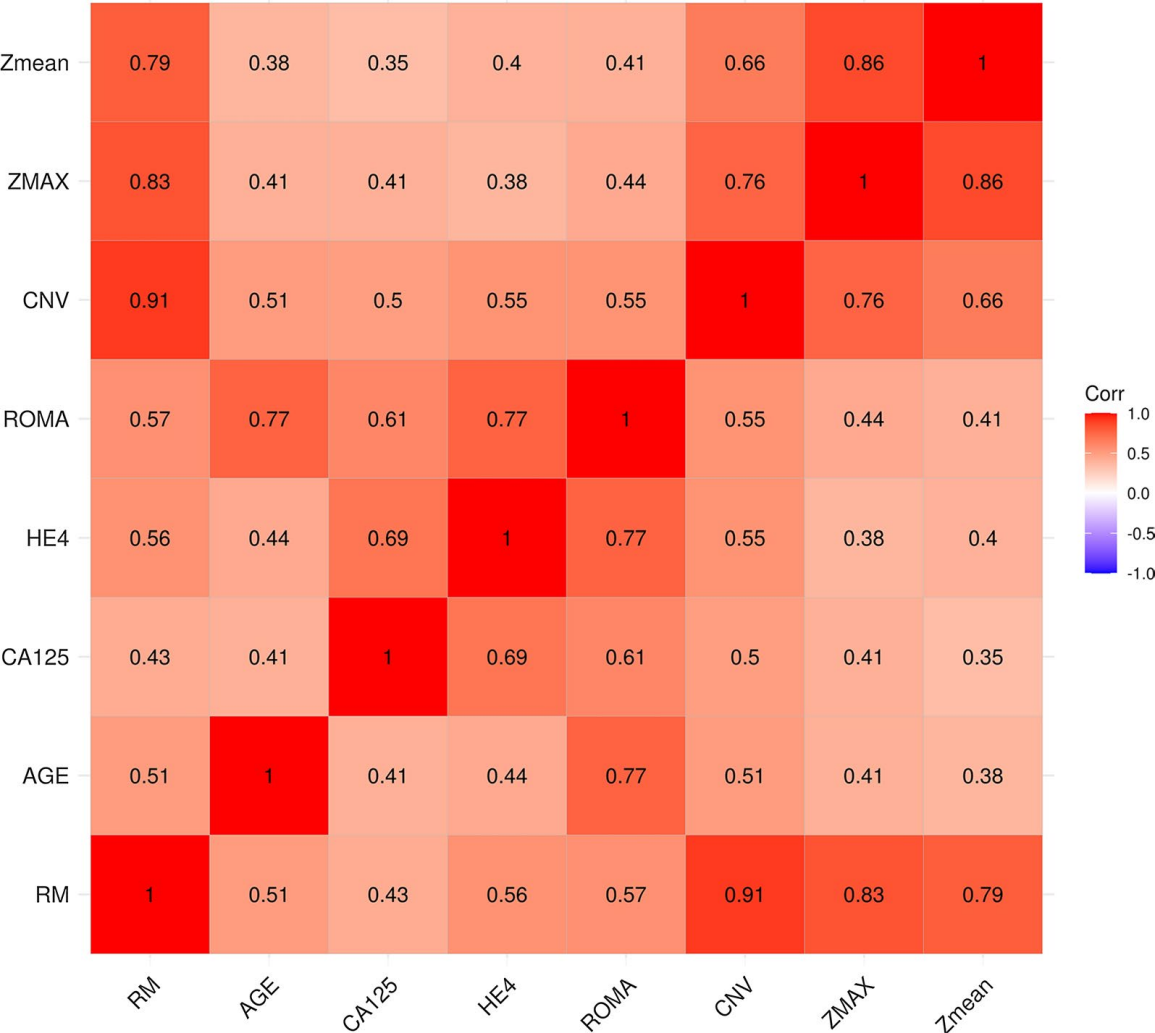
Spearman correlation analysis of variables

Spearman correlation was used to investigate the relationship between tumor markers and LCWGS index. As shown in Fig. 3 and Table 3, all indexes were statistically correlated (*P* < 0.01). However, the correlation between traditional tumor markers and LCWGS index was weak (r value range from 0.38 to 0.77). The weak correlation showed that RM and ROMA could be used as a complementary in the diagnosis of pelvic malignant mass.

**Table 3:** Correlation analysis of laboratory index

	RM	AGE	CA125	HE4	ROMA	CNV	ZMAX	Zmean
RM	1	0.51**	0.43**	0.56**	0.57**	0.91**	0.83**	0.79**
AGE	0.51**	1	0.41**	0.44**	0.77**	0.51**	0.41**	0.38**
CA125	0.43**	0.41**	1	0.69**	0.61**	0.50**	0.41**	0.35**
HE4	0.56**	0.44**	0.69**	1	0.77**	0.55**	0.38**	0.40**
ROMA	0.57**	0.77**	0.61**	0.77**	1	0.55**	0.44**	0.41**
CNV	0.91**	0.51**	0.50**	0.55**	0.55**	1	0.76**	0.66**
ZMAX	0.83**	0.41**	0.41**	0.38**	0.44**	0.76**	1	0.86**
Zmean	0.79**	0.38**	0.35**	0.40**	0.41**	0.66**	0.86**	1

*p < 0.05, **p < 0.01

### Comparison of the diagnostic value of LCWGS and traditional tumor markers

**Fig. 4:**
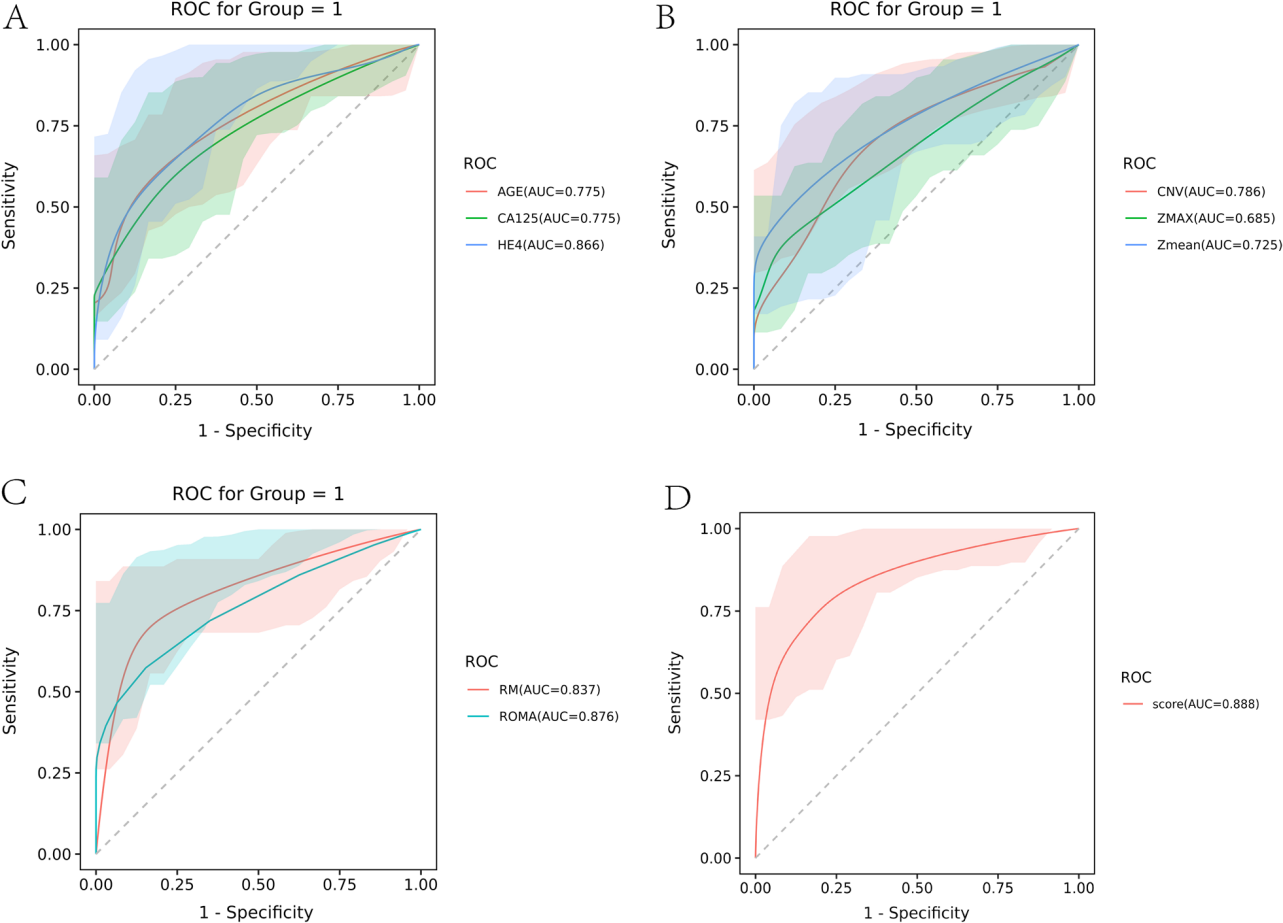
ROC of ROMA and RM. The ROC included **A** Age, CA125, HE4; **B** CNV, Zmax, Zmean; **C** ROMA and RM; **D** RM combine CA125 and HE4. There were no significant differences in AUC of ROMA and RM (Delong test: *p* = 0.476).1: malignancy mass

Firstly, we evaluated the diagnostic value of single index in the reasearch subjects. The AUC of CA125 and HE4 was 0.775 and 0.866 respectively. HE4 showed better diagnostic accuracy than other markers. Then the integrated indexes were evaluated. The AUC of ROMA and RM was 0.876 and 0.837, respectively. And the AUC of RM combine CA125 and HE4 was 0.888. Both ROMA and RM showed higher diagnostic accuracy than single index. However, no significant difference was found between ROMA and RM (Delong test: P = 0.476), which indicated that ROMA and RM had similar diagnostic value between ovarian cancers and benign diseases. With the cutoff of 0.085, the sensitivity and specificity of ROMA was 0.684 and 0.909 respectively. With the cutoff of 1.25, the sensitivity and specificity of ROMA was 0.895 and 0.773 respectively (Fig. 4 and Table 4).

**Table 4:** Result comprision of ROC

Variable	AUC	Stderr	CI	Cutoff	Specificity	Sensitivity
AGE	0.775	0.061	0.655–0.894	48.5	0.545	0.947
CA125	0.775	0.062	0.653–0.897	55.535	0.818	0.684
HE4	0.866	0.055	0.758–0.974	55.93	0.841	0.789
ROMA	0.876	0.045	0.788–0.964	0.085	0.909	0.684
CNV	0.786	0.057	0.675–0.898	4.5	0.614	0.842
ZMAX	0.685	0.069	0.549–0.822	8.305	0.409	0.947
Zmean	0.725	0.07	0.588–0.863	4.355	0.705	0.789
RM	0.837	0.051	0.736–0.938	1.25	0.773	0.895

### Validation study

In the validation set, there were 15 patients enrolled in the malignant group and 9 patients enrolled in the benign group. The histology of malignant group in validation study included ovarian high-grade serous adenocarcinoma (n = 6), Mucinous cystadenocarcinoma (n = 3), Borderline serous cystadenoma (n = 6). Among 15 malignant patients, 3 patients were at stage II, 6 patients were at stage III and 6 patients were at stage IV. Significant differences of age, marriage, childbirth and menopause status were found between the two group. In the validation cohort, the AUCs of ROMA and RM were 0.978 and 0.867 respectively. RM showed better diagnostic value than ROMA. ALL data about the validation study in listed in supplement Table 2.

## Discussion

As the second highest incidence and mortality of female reproductive system tumor following cervical cancer, ovarian cancer has the early clinical presentation that are difficult to be differentiated from digestive tract diseases, such as bloating or abdominal pain [15, 16]. When ovarian cancer develops and spreads to the abdominal cavity, abdominal mass may appear [17]. Therefore, distinguishing between benign and malignant abdominal masses is very important for the early diagnosis of ovarian cancer.

Oncogenesis involves many types of genomic variation, such as point mutation, copy number variation and gene fusion [18]. Tumors are different from genetic diseases, and their genomic variation is frequently acquired [19]. The development of ovarian cancer is a complex process involving the changes of DNA, RNA, and proteins [20, 21]. The abnormal DNA of cancers could release from cancer tissues and be detected in blood samples in the form of cell free DNA [22]. Therefore, the detection of CNVs would be a promising method for the identification of malignant abdominal masses.

In this study, we evaluated whether CNVs detected by LCWGS platform could accurately predict the existence of malignancy. In our study cohort, the number of patients with malignant (43 cases) was higher than the patients with benign disease (19 cases). In addition, the patients with malignant disease were older than patients with benign disease. The difference in age distribution between malignant and benign patients would have impact on the level of tumor markers, however, the impact of age on CNVs was little. Our results showed that, chromosome variation could be detected in cell free DNA in patients with malignancy. However, only a few cases with malignant mass showed trisomy or monosomy. Despite that chromosome instability was common in tumor cells, owing to the low concentration of tumor derived cell free DNA, detection of trisomy or monosomy might lack sensitivity for clinical diagnosis [23]. We set our detection target to CNVs at the resolution of 5 MB. With this strategy, more chromosome instabilities could found in the subjects, however, the specificity might reduce. To solve this problem, we extracted more indexes from the LCWGS results and a healthy cohort was used to calibrate our results. Our results indicate that LCWGS based indexes were significantly different between patients with malignant and benign diseases and closely related to FIGO Stage, which would be valuable in the diagnosis of malignant mass. The diagnostic value of LCWGS based indexes were evaluated by ROC curve. Despite that CNV counts, Zmax and Zmean were useful for the diagnosis of malignant mass, however, the AUCs were less than 0.80. An integrated RM index which is calculated by CNV and Zmean and calibrated by a healthy cohort, showed better diagnostic performance with a AUC of 0.837. With the cut-off value of 1.25, RM is highly sensitive in the detection of malignant mass with all stage.

Both CA125 and HE4 were the most widely used markers in ovarian cancer diagnosis [24]. In our study, CA125 and HE4 showed significant difference between the malignant mass and benign disease, which is consistent with previous reports. In 2009, Moore proposed ROMA as a new algorithm. He correlated HE4 and CA125 levels with menopausal status, which was defined as 6 months of menopause without menstruation or clinical symptoms. The ROMA corresponds to the predicted probability [PP], expressed as a percentage [14]. The sensitivity of ROMA for ovarian cancer diagnosis varies from 75 to 97%, however, the detection of early stage malignancy was still a problem [25–27]. We compared the diagnostic value between RM and ROMA, despite that ROMA showed higher AUC than RM, however, the difference was not statistically significant. The sensitivity of RM (0.895) is superior to that of ROMA (0.684), while the specificity of RM (0.773) is inferior to that of ROMA (0.909). The CA125 and HE4 were correlated with LCWGS based index. However, the correlation was weak. Therefore, RM and ROMA could be used as a complementary in the diagnosis of pelvic malignant mass.

To validate our results, another 24 patients from Sun Yat-Sen University Cancer Center were recruited with the same inclusion criteria and tested by LCWGS. Our results showed that the LCWGS strategy was still a useful tool in the discrimination of malignant and benign diseases and showed better diagnostic performance than ROMA. In the validation study, the patients with malignant disease were at advanced stage, which would explain that why the AUC of RM is higher than that in the training study.

Low specificity of RM may originate from the bio-informatics pipeline in LCWGS. All CNVs in whole genome were used for further analysis. Ovarian cancers showed specific gain or loss of chromosomes in tissues as demonstrated by other studies, however, there was no widely accepted specific CNVs in cell free DNAs [28]. Further studies should be developed and focus on ovarian cancer specific CNVs to improve the diagnostic specificity. In addition, the increase of sequencing depth would be helpful in increasing the diagnostic value. Further studies could try to ascertain the sequencing depth regarding with the cost and effect.

A limitation of this study was that the number of patients was small. A larger sample size is needed to validate our findings, and to conduct further studies on different FIGO stages of ovarian cancer or in patients with pre- and post-menopause.

In conclusion, our study provided a new methodology with high accuracy for the diagnosis of ovarian cancers, which could be a supplement to the existing diagnostic methods.

## Supplementary Information

**Additional file 1:** Supplement Table 1. CNVs and Z scores in all subjects.

**Additional file 2:** Supplement Table 2. Comparison of laboratory index in validation group.

## Data Availability

The data and material in our studies were availability.

## Abbreviations

LCWGS: Whole genome sequencing
CNVs: Copy number variants
CNA: Copy number alterations
RM: Risk of malignancy
ROMA: Risk of ovarian malignancy algorithm
ROC: Receiver Operating Characteristic Curve
Zmean: Mean of Z-scores
Zmax: The max of Z-scores
AUC: Area under curve
CA125: Cancer antigen 125
HE4: Human epididymis protein 4
NGS: Next generation sequencing
